# Novel fabrication of a robust superhydrophobic PU@ZnO@Fe_3_O_4_@SA sponge and its application in oil-water separations

**DOI:** 10.1038/s41598-017-17761-9

**Published:** 2017-12-13

**Authors:** Viet-Ha Thi Tran, Byeong-Kyu Lee

**Affiliations:** Department of Civil and Environmental Engineering, University of Ulsan, Nam-gu Daehak-ro 93, Ulsan, 680-749 Republic of Korea

## Abstract

We report a novel superhydrophobic material based on commercially available polyurethane (PU) sponge with high porosity, low density and good elasticity. The fabrication of a superhydrophobic sponge capable of efficiently separating oil from water was achieved by imitating or mimicking nature’s designs. The original PU sponge was coated with zinc oxide (ZnO), stearic acid (SA) and iron oxide particles (Fe_3_O_4_) *via* a facile and environmentally friendly method. After each treatment, the properties of the modified sponge were characterized, and the changes in wettability were examined. Water contact angle (WCA) measurements confirmed the excellent superhydrophobicity of the material withhigh static WCA of 161° andlow dynamic WCA (sliding WCA of 7° and shedding WCA of 8°). The fabricated sponge showed high efficiency in separation (over 99%) of different oils from water. Additionally, the fabricated PU@ZnO@Fe_3_O_4_@SA sponge could be magnetically guided to quickly absorb oil floating on the water surface. Moreover, the fabricated sponge showed excellent stability and reusability in terms of superhydrophobicity and oil absorption capacity. The durable, magnetic and superhydrophobic properties of the fabricated sponge render it applicable to the cleanup of marine oil spills and other oil-water separation issues, with eco-friendly recovery of the oil by simple squeezing process.

## Introduction

With the increasing use of fossil fuel and the global population growth, oil spill accidents occurring during oil utilization and transport processes have adversely impacted the environment. For example, the Deep-water Horizon oil spill (2010) in Mexico was considered the world’s largest accidental release of oil into marine water in history, causing significant environmental damage associated with the discharge of nearly five million barrels of oil and the death of eleven people^1^. More recently, a pipeline spill (2016) in North Dakota, USA, leaked 4200 barrels of oil, and up to 5.4 miles of a creek was heavily polluted^2^. Therefore, the separation of oil from water has received great attention from scientists and engineers because of the increase in oil pollution events worldwide. However, it is very difficult to collect or separate spilled oil from bodies of water. Thus, oil companies have spent large sums to clean up oil spills. Many remediation processes, such as electrochemical methods^3,4^, controlled burning^5,6^, membrane filtration^7,8^, chemical dispersants and biological agents^9,10^, have been developed to clean up oil pollution. However, most of these methods suffer from high operation costs, low efficiency and, in some cases, the creation of secondary pollutants.

Consequently, the developments of advanced materials capable of selectively separating oil from bodies of water in oil spill areas are highly desirable. Currently, superhydrophobic materials with a static water contact angle (WCA) higher than 150° and a dynamic WCA less than 10° have attracted attention due to their unique super-antiwetting, self-cleaning properties and their potential for use in practical applications^11–14^, including oil-water separation. Our relevant literature survey revealed that an increasing number of studies with the topics of “oil-water separation” and “superhydrophobic surface” were published from 2007 to 2016 (Fig. S1). This clearly demonstrates the focus of investigations on durable superhydrophobic materials for application to the proper separation of oil and water and the clean-up of spilled oils.

In nature, water repellence and superhydrophobic phenomena are frequently observed, for example, lotus leaves (*Nelumbo buciferea*) with nano/micro structures, Ramee leaves (*Boehmeria nivea*) and Chinese watermelon (*Citrullus lanatus*) with microstructures on their surfaces all exhibit hydrophobic properties. The term biomimetic is defined as actions to imitate or mimic nature. The rapidly increasing interest in the biomimetic field is creating a new current trend in research, which includes mimicking natural surface structures to develop desirable materials, devices and processes. Artificial superhydrophobic surfaces are developed based on mimicking natural superhydrophobic phenomena by employing two approaches: (i) utilization of micron- or nanometer-scale surface roughness and (ii) application of chemical hydrophobicity. While the initial surface is hydrophilic, proper control of the roughness and a surface treatment or coating are required to switch from a hydrophilic state to a superhydrophobic state. Generally, superhydrophobic surfaces have been developed in the form of fabrics^15,16^, meshes^17^, films^18^ and 3D porous materials^19^. Among these materials, commercially available polyurethane (PU) sponge, which has a high porosity, light weight and good elasticity, is a promising substrate for the preparation of a superhydrophobic material for oil-water separation and oil absorption. However, the PU sponge is hydrophilic and can easily absorb both oil and water. Therefore, researchers have tried various methods to change the wettability of the original PU sponge by increasing the surface roughness with SiO_2_/graphene oxide nanohybrids^20^ or changing the chemical functional groups with carbon nanotubes/poly(dimethylsiloxane)^21^ or SiO_2_/poly(tetrafluoroethylene)^22^. However, these materials are very expensive, non-biodegradable and harmful to the environment. Furthermore, most researchers have focused on only one approach for fabricating superhydrophobic PU and, to the best of our knowledge, no studies have combined both approaches for attaining superhydrophobicity.

In this study, we report the preparation of a novel, magnetic, durable and superhydrophobic composite material based on a commercial PU sponge *via* a facile method. The surface of the fabricated material clearly mimics natural superhydrophobic phenomena. The hydrophobicity was first derived from the microstructure, which was grown on the initial surface and was similar to that observed on the natural surface of Ramee leaves or Chinese watermelon. For this first approach towards engineering a suitable surface roughness, a zinc oxide (ZnO) coating layer was selected due to its superior abilities, which include easily controlled of structure growth, low cost and environmental non-toxicity^15,23^. After a facile ZnO coating step performed with a commercial microwave, the wettability of the PU sponge was transformed from a hydrophilic state to a hydrophobic state. In the second step of engineering, to achieve the surface chemical hydrophobicity observed for the wax surface of the lotus leaf, stearic acid (SA) (Fig. S2) - a long-chain fatty acid - was used as a modifier to tune the surface wettability from hydrophobic to superhydrophobic. The functionalized sponge should also exhibit magnetic responsivity for the treatment of oil floating on the composite surface due to the addition of Fe_3_O_4_ particles to the surface. The fabricated PU@ZnO@SA@Fe_3_O_4_ sponge was then tested in two experiments: (i) selective absorption of oil floating on water and (ii) separation of oil from a mixture with water. The oil sorption capacity and separation efficiency of the sponge were investigated for hexane, toluene, dichloromethane, gasoline, soybean oil, diesel engine oil and vacuum pump oil. The durability and reusability of the fabricated sponge were also tested. Because of its robust superhydrophobicity and good mechanical stability, the as-prepared PU@ZnO@Fe_3_O_4_@SA sponge ranks as a promising material for practical application to the sorption and recovery of oil from water.

## Results

### Characterization of the fabricated materials

#### Surface morphology

**Figure 1:**
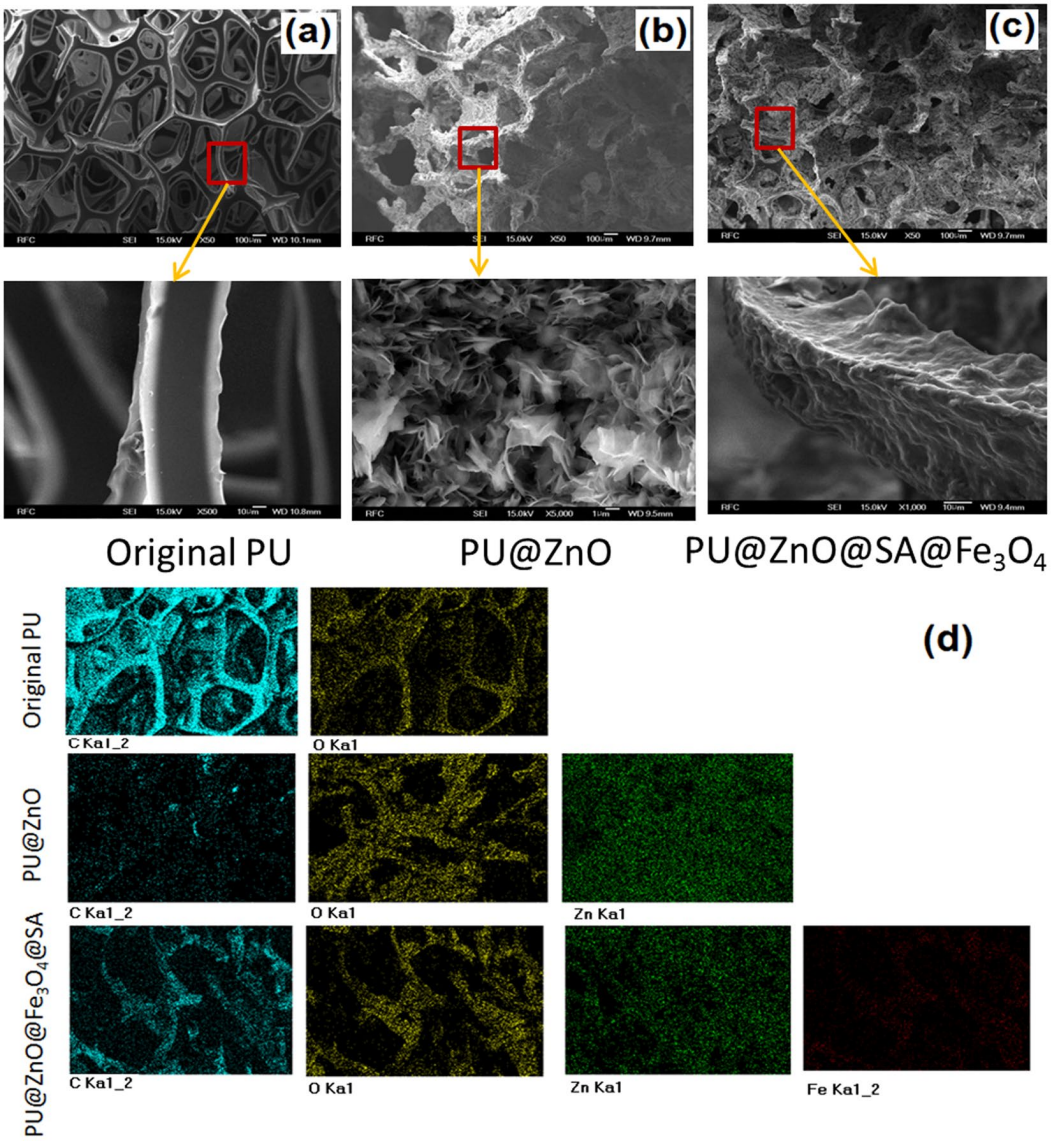
SEM images of (**a**) the original PU, (**b**) PU@ZnO, (**c**) PU@ZnO@Fe_3_O_4_@SA, and (**d**) mapping images of these sponges.

The surface roughness is the first factor that should be considered in achieving a robust superhydrophobic state. Before modification, the PU substrate had a three-dimensional porous structure with a relatively smooth surface and an open cell structure (Fig. 1a). As described above, in the first step of the modification process, ZnO was grown on the original PU sponge to afford a PU@ZnO sponge. In this step, high-density ZnO flakes were deposited on the PU substrate, which roughened the surface of the PU scaffold (Fig. 1b). This fabrication method, performed with a commercial microwave oven, was very useful for forming a relatively uniform ZnO structure on the PU surface. Furthermore, the use of a commercial microwave as the heating source to grow the structured ZnO shortened the fabrication time, reduced the specific equipment requirement and lowered the energy usage, thus rendering it an eco-friendly fabrication method.

In addition to the surface roughness, the chemical hydrophobicity of the material is an equally important factor. Our aim was to achieve a superhydrophobic material. Thus, SA was chosen to modify the sponge because it is a low surface energy material. After treatment with SA@Fe_3_O_4_-ethanol and SA-ethanol solutions, a PU@ZnO@Fe_3_O_4_@SA sponge was obtained (Fig. 1c). By adding Fe_3_O_4_ particles, the fabricated sponge was imparted with magnetically responsive properties, which is helpful for applications in floating oil absorption. The SA molecules formed a dense self-assembled monolayer on the ZnO surface as a result of the strong chelating bonds formed between the carboxylates and Zn atoms on the surface^24^. Therefore, the structure of the ZnO layer could no longer be clearly observed due to the fatty acid top-coating layer. Nevertheless, the achieved roughness of the PU@ZnO@Fe_3_O_4_@SA surface (Fig. 1c) was still much higher than that of the untreated PU sponge (Fig. 1a). This roughness was beneficial not only for imparting superhydrophobicity but also for increasing the surface area contact with oil, which improved the oil affinity of the material. The addition of Fe_3_O_4_ particles could not be observed in the SEM image due to their random deposition. However, the presence of the particles was confirmed by other analysis techniques, including mapping, XPS and XRD. The typical X-ray mapping results presented in Fig. 1d provided information about the elemental distributions in the samples. The distributions of carbon (original content of the PU sponge), oxygen, zinc and iron on the material surface were uniform, which further supports the presence of the desired components on the fabricated material.

The initial porosity of reticulated foams is critical when designing a custom component or product. The term “porosity” is evaluated by the pores per inch (PPI) value, which is designated as the number of pores in one linear inch. The calculated PPI value of the original PU was approximately 60, corresponding to homogenous and uniform cells. The inspection of Fig. S3 revealed that the pore sizes and PPI values of the sponges were not greatly changed after modification compared with the initial values. This confirmed that the extra coating layers did not exert significant pore-blocking effects on the fabricated sponge.

#### XRD analysis and magnetic properties

**Figure 2:**
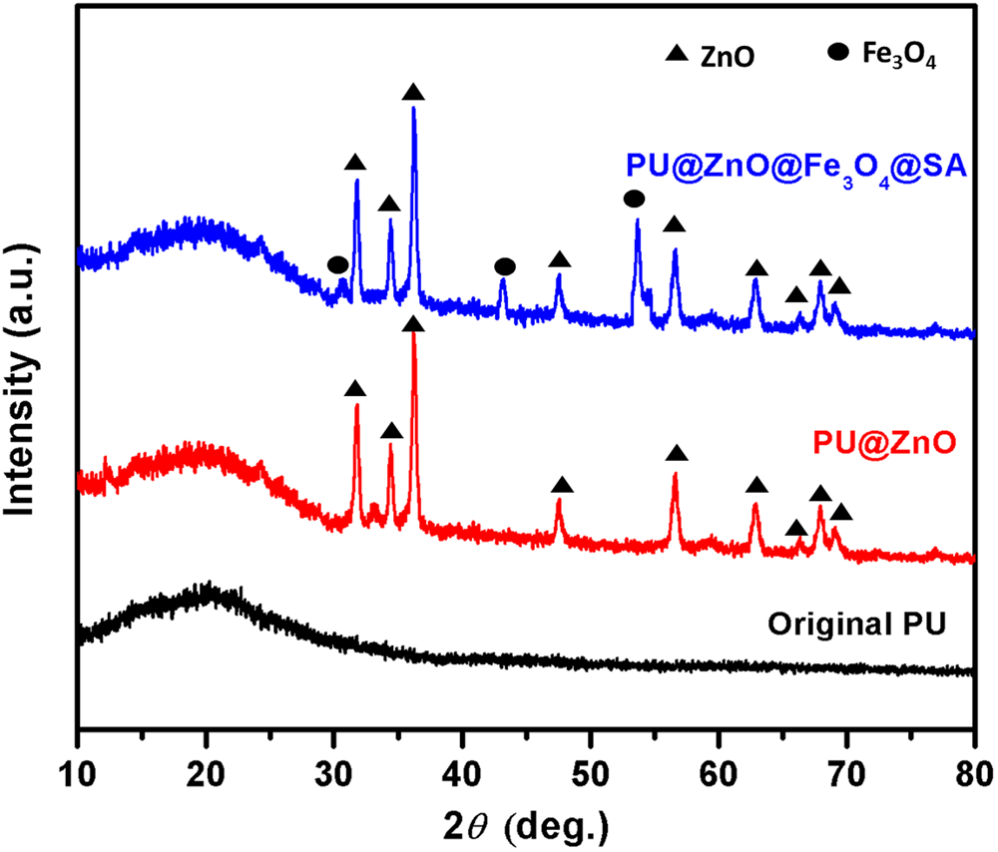
XRD pattern of sponges samples.

Figure 2 shows the XRD patterns of the original PU, PU@ZnO and PU@ZnO@Fe_3_O_4_@SA sponges. The broad peak detected in the XRD curves of all the samples indicates the low degree of crystallinity of PU. Compared to the XRD pattern of the original PU, the XRD patterns of PU@ZnO and PU@ZnO@Fe_3_O_4_@SA contain additional peaks at 2*θ* = 32.01°, 34.17°, 36.20°, 47.35°, 56.62°, 62.92°, 66.36°, 68.03° and 69.09°. These peaks correspond to the structure of ZnO according to the values of the Joint Committee on Powder Diffraction Standard (JCPDS) No. 36–1451^15,23,25^. Characteristic peaks at 2*θ* = 30.1°, 43.1° and 53.5° are observed in the PU@ZnO@Fe_3_O_4_@SA sponge sample. These peaks fit well with the Fe_3_O_4_ patterns (JCPDS No.65–3107) reported in previous studies^26,27^. The absence of any signals of impurities in the XRD patterns confirms the high purity level of the fabricated samples.

Figure S4 shows the magnetic hysteresis loops of the fabricated sponge samples. The PU@ZnO@Fe_3_O_4_@SA sponge became magnetic with a saturation magnetization following the addition of Fe_3_O_4_ particles, and this magnetic property was still maintained even after 100 cycles. These results showed that the magnetic strength of the fabricated sponge was sufficient for it to be easily manipulated and guided by a magnet for the treatment of oil floating on the water surface.

#### FT-IR and XPS analysis

**Figure 3:**
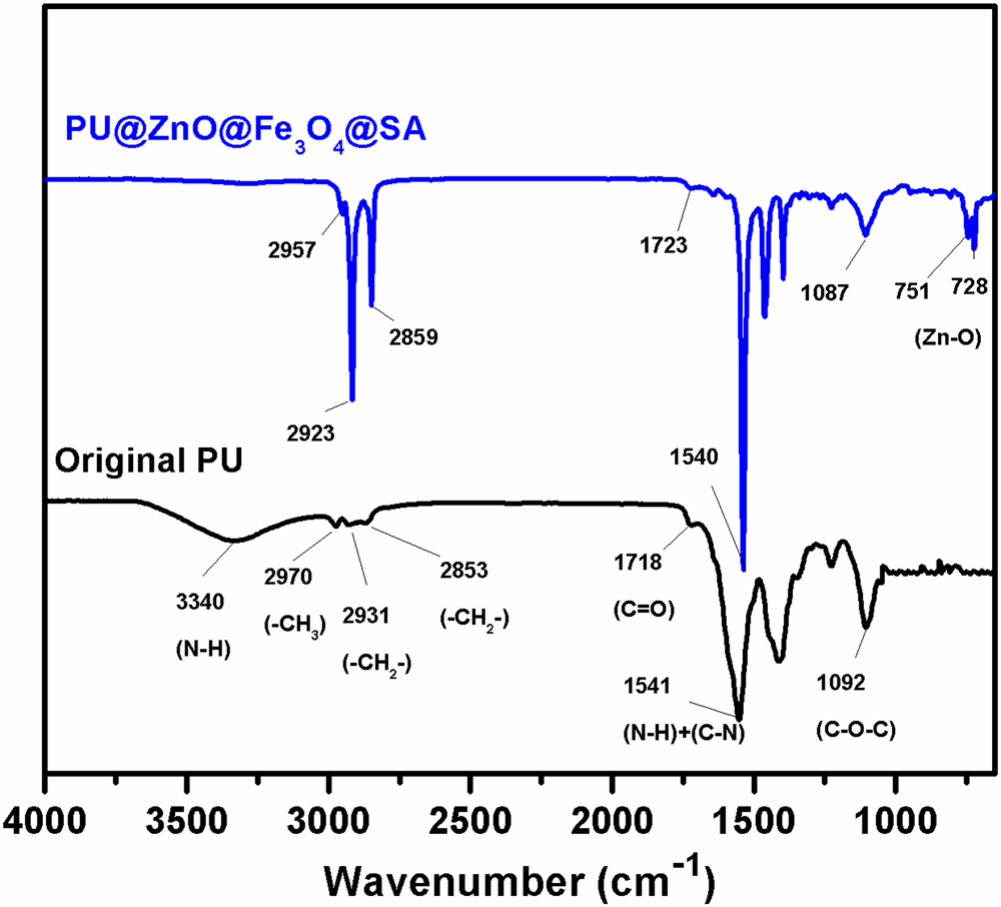
FT-IR spectra of the original PU and PU@ZnO@Fe_3_O_4_@SA sponges.

FT-IR spectroscopy was used to investigate the possible interactions between the surface of the original sponge and the other functional groups. The FT-IR spectra of the original PU and PU@ZnO@Fe_3_O_4_@SA sponges are displayed in Fig. 3. In the original PU sponge, the bands at 3340 cm^−1^ and 1541 cm^−1^ are consistent with the stretching of N-H bonds, which is consistent with the characteristic bands of urethane and urea groups^28^. The vibrations at 2970, 2931, and 2853 cm^−1^ are associated with the -CH_3_ asymmetric stretching, -CH_2_- symmetric stretching and -CH_2_- asymmetric stretching, respectively. The other characteristic bands of PU were also observed at 1718 cm^−1^, corresponding to C = O stretching vibrations^29^, and 1092 cm^−1^, corresponding to C-O-C symmetric stretching vibrations^30^. All of these characteristic peaks confirmed that the original sponge was a typical kind of PU. Compared with the original PU sponge, the peaks at 751 and 728 cm^−1^ observed for PU@ZnO@Fe_3_O_4_@SA were attributed to ZnO stretching modes^31–33^. In addition, the IR vibrations of -CH_3_ and -CH_2_- in the PU@ZnO@Fe_3_O_4_@SA sponge exhibited an obvious increase in intensity and thus provide further evidence that SA was anchored on the surface of the original PU sponge.

**Figure 4:**
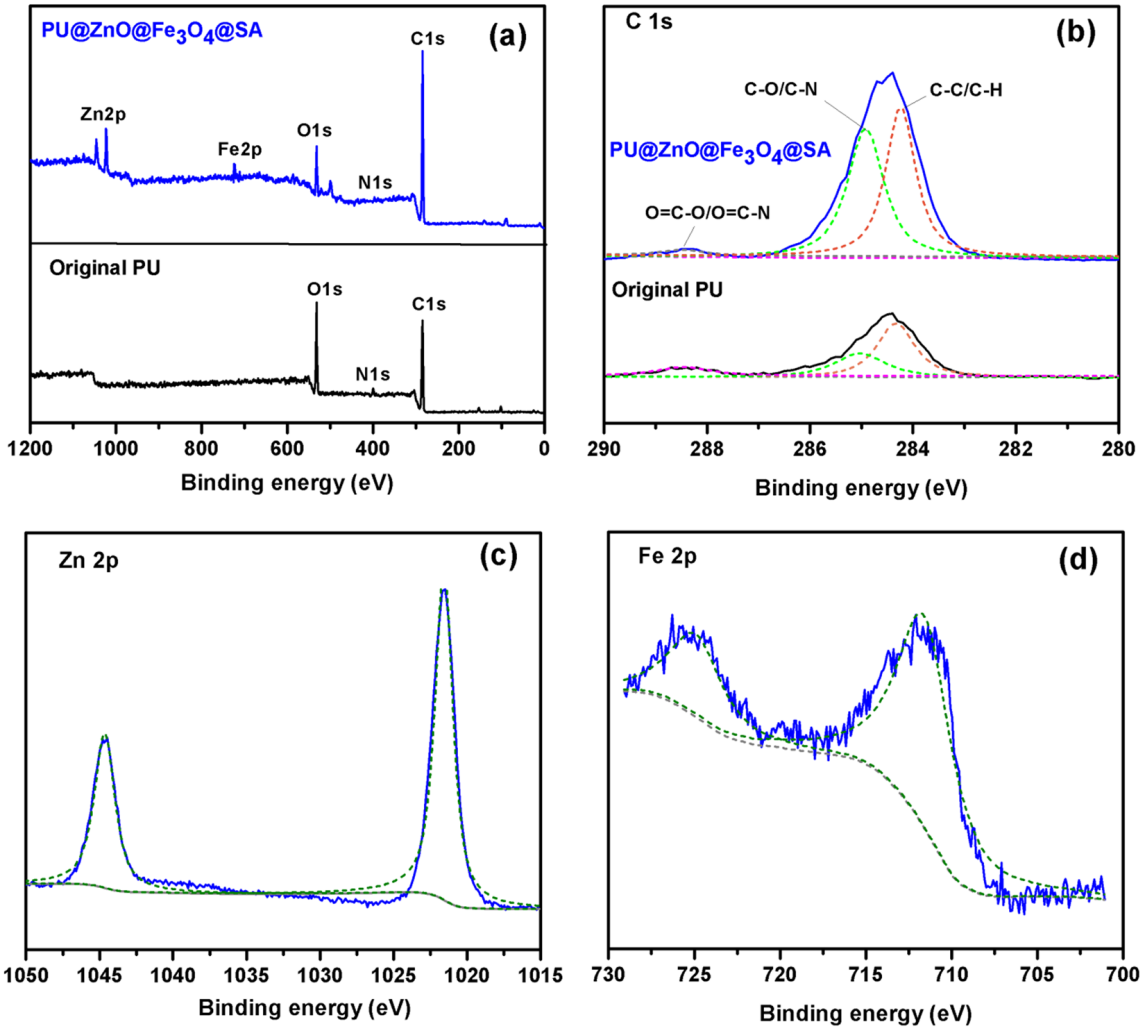
XPS data of the original PU and PU@ZnO@Fe_3_O_4_@SA sponges: (**a**) survey spectra, (**b**) C 1 s spectra; and fitting peak of (**c**) Zn 2p and (**d**) Fe 2p spectra of the PU@ZnO@Fe_3_O_4_@SA sponge.

Figure 4 depicts the XPS spectra of the original PU and PU@ZnO@Fe_3_O_4_@SA sponges. Compared with the original PU sponge, two new peaks of Zn 2p and Fe 2p appeared, and the peak intensity of C 1 s clearly increased in the PU@ZnO@Fe_3_O_4_@SA sponge (Fig. 4a). Figure 4b shows the corresponding C 1 s XPS spectra of the sponges. The observed peaks correspond to the C-C/C-H bonds (284.23 eV), C-O/C-N bonds (285.08 eV) and O = C-O/O = C-N bonds (288.47 eV) present in both samples. The clear increase in the intensity of the C-C/C-H bonds in the modified sample is solid evidence for the presence of the long carbon chains of SA. The observation of Zn 2p and Fe 2p peaks (Fig. 4c and d) proves the formation of ZnO and Fe_3_O_4_ on the PU sponge surface. Combined with the XRD and FT-IR results, the XPS results further confirmed that the original PU sponge was thoroughly coated with a ZnO layer, a SA layer and Fe_3_O_4_ particles.

#### Wettability measurements

**Figure 5:**
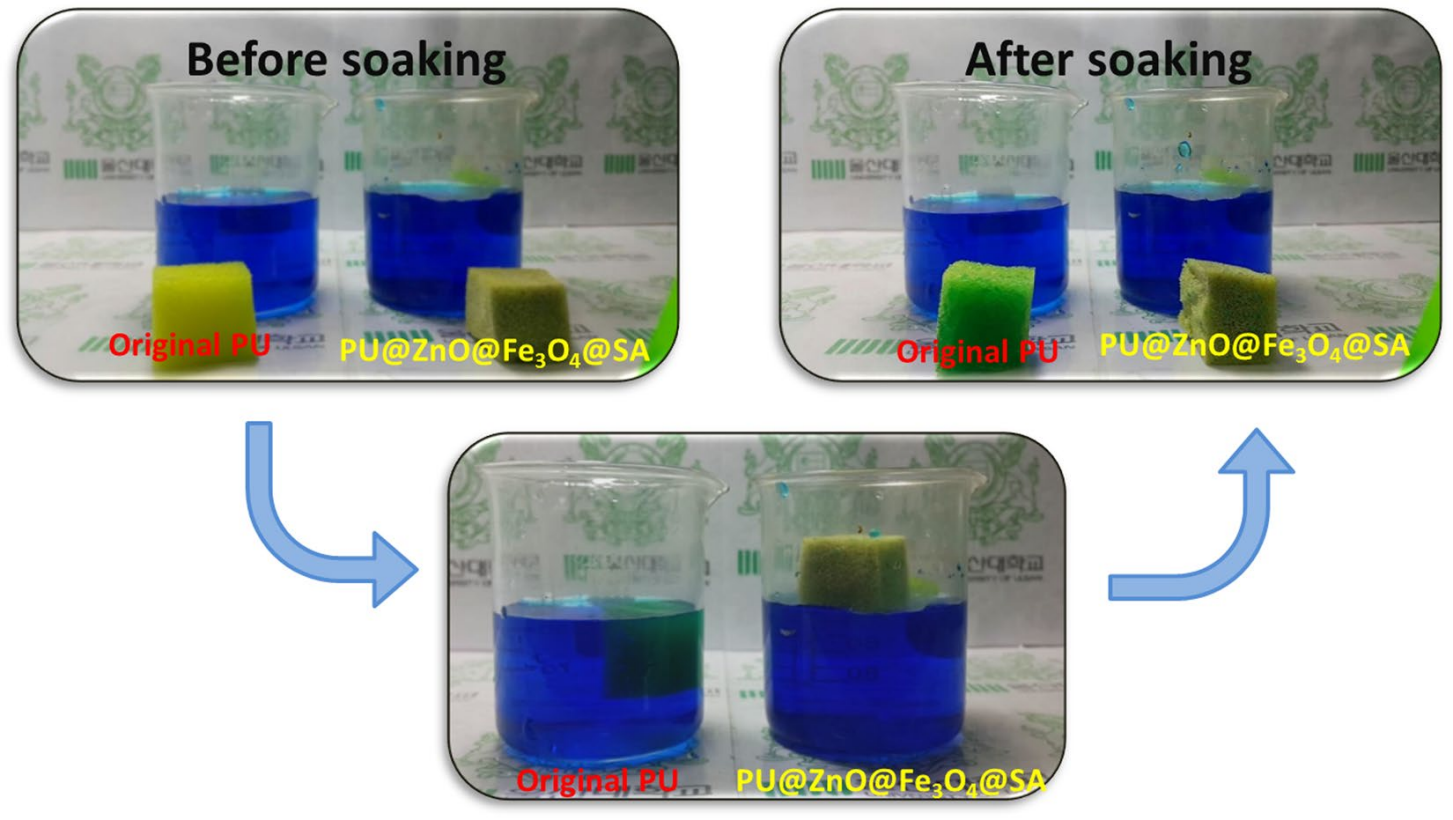
Soaking experiment with dye solution and different wetting behaviors of the original PU and PU@ZnO@Fe_3_O_4_@SA sponges.

The wettability of the original PU was compared with that of the fabricated PU@ZnO@Fe_3_O_4_@SA sponges *via* a soaking test, as shown in Video S1. The two sponges were immersed in an aqueous solution of methylene blue dye with a concentration of 0.1 M. Figure 5 also depicts photographs of the hydrophilic PU sponge, which quickly sank in the dye solution, while the superhydrophobic PU@ZnO@Fe_3_O_4_@SA sponge floated on the top of the same dye solution.

**Figure 6:**
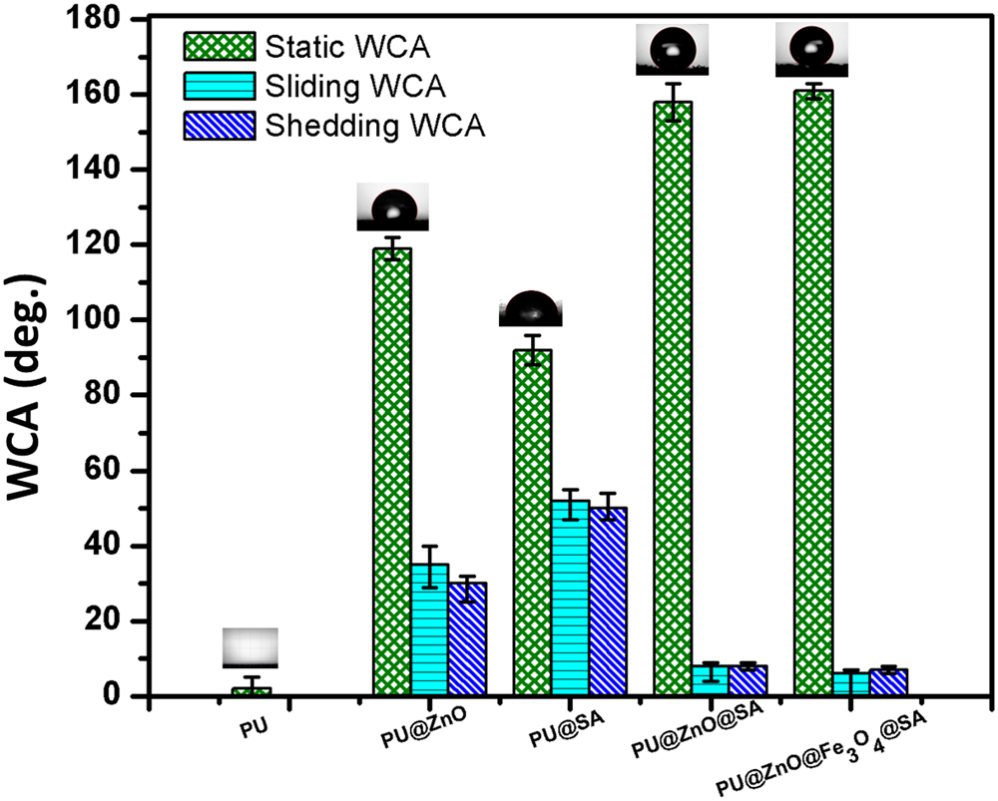
WCAs of the original PU, PU@ZnO, PU@SA, PU@ZnO@SA and PU@ZnO@Fe_3_O_4_@SA.

For scientific analysis, the WCA measurement method was used to examine the surface wettability of the sponge. The static WCA measurement alone is does not provide a reliable evaluation due to the macroscopic nature of the sponge surfaces. Thus, the wettability of the sponge surface was also evaluated by dynamic WCA (including sliding WCA and shedding WCA) measurements. Theoretically, a surface with a static WCA lower than 90° is termed a hydrophilic surface. In contrast, surfaces with static WCAs greater than 90° and 150° are called hydrophobic and superhydrophobic surfaces, respectively. Furthermore, a superhydrophobic surface also requires a dynamic WCA lower than 10°. The hydrophobicity of the surface can be attributed to two factors: the surface topography and the surface energy. We verified the role of these two factors on the wetting state of the synthesized PU@ZnO@Fe_3_O_4_@SA sponge by comparing the measurement results of the WCA of the following samples: (i) the original PU, (ii) PU@ZnO, (iii) PU@SA, (iv) PU@ZnO@SA and (v) PU@ZnO@Fe_3_O_4_@SA (Fig. 6).

The results showed that the static WCA increased from close to 0° on the original hydrophilic PU sponge surface to 119° for the PU@ZnO sponge. The Wenzel and Cassie-Baxter models, which are used to describe the wetting of a rough surface, can also be used to explain the improved static WCA for the PU@ZnO sponge. As mentioned in section 3.1.1, after the first coating step, ZnO grew on the PU sponge surface in rough flake structures. Due to the high roughness of these structures, air pockets are trapped in the rough cavities, which help to increase the surface hydrophobicity. On the other hand, a coating of steric acid can also increase the hydrophobicity of the PU@SA surface. The long-chain, hydrophobic alkyl groups of SA (Fig. S2) were introduced to achieve a low surface energy; thus, the static WCA of the PU@SA sample exhibited a value of 92°. However, the static WCA values of these two sponges indicated the achievement of a hydrophobic state, and a superhydrophobic state was still not attained.

The data shown in Fig. 6 indicated that a superhydrophobic state was only attained when both ZnO and SA layers were introduced. The ZnO flakes first provided the necessary roughness features. After that, the polar, hydrophilic head of SA bound to ZnO, while the long, hydrophobic tail chain of SA was exposed outside, which induced superhydrophobicity^34–37^. The increased roughness and the lowered surface energy clearly exerted simultaneous effects on the superhydrophobic state of the sponge. The static WCAs of PU@ZnO@SA and PU@ZnO@Fe_3_O_4_@SA were 158° and 161°, respectively. The addition of Fe_3_O_4_ particles also had no significant effect on the wettability of the sponge surface. Furthermore, the dynamic WCAs of these two samples were lower than 10° [sliding WCA = 8° and shedding WCA = 7° (Video S2)], which strongly confirmed the excellent superhydrophobicity of the fabricated PU@ZnO@Fe_3_O_4_@SA sponge.

Interestingly, the fabricated PU@ZnO@Fe_3_O_4_@SA sponge showed both superhydrophobicity and oleophilicity properties. An experiment to demonstrate these properties was carried out and is shown in Video S3. The wettability of the original PU sponge was completely transformed after modification. The original PU sponge was easily wetted by both water and oil drops. In contrast, water drops could not be absorbed by the PU@ZnO@Fe_3_O_4_@SA sponge, whereas oil could still penetrate. Based on this observation, the use of the fabricated PU@ZnO@Fe_3_O_4_@SA sponge for oil-water separation was proposed and is described in the next section.

### Selective oil-water separation

Based on the feasibility test for the water and oil absorption capacity shown in Video S3, the superhydrophobic PU@ZnO@Fe_3_O_4_@SA sponge could be considered a promising material for selective oil-water separation applications. Two different routes for using the fabricated PU@ZnO@Fe_3_O_4_@SA sponge to separate oil and water were investigated, as described in the experimental section.

**Figure 7:**
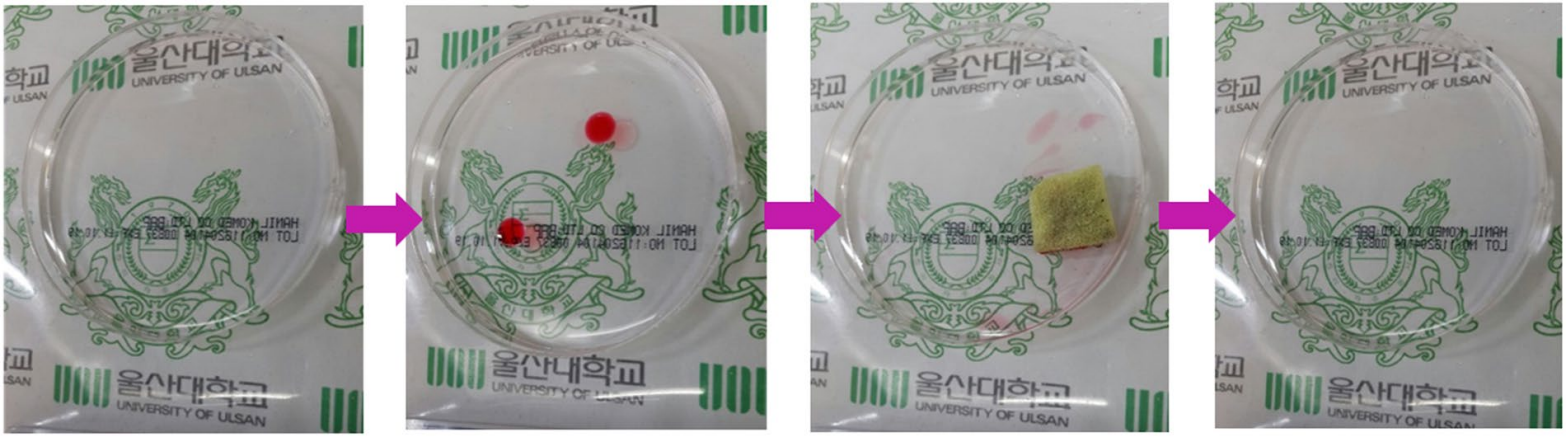
Photographs of using PU@ZnO@Fe_3_O_4_@SA to remove floating oil.

The first experiment was conducted on floating oil and is shown in Fig. S5, Fig. 7 and Video S4. By using an external magnet to place and control the direction of the fabricated sponge on the surface of the oil-water mixture, the floating oil in the polluted regions, shown in red color, was rapidly absorbed into the sponge, thereby purifying the water underneath. The sponge was removed from the solution when no sign of the dyed oil could be seen on the water surface. The absorbed oil was easily collected by squeezing due to the elasticity of the PU sponge. The proposed oil collection method is more environmentally friendly, faster and more cost efficient than other reported methods, such as burning off^6^ or heat treatment^38^ processes. Therefore, the fabricated sponge is applicable for oil spill cleanup.

**Figure 8:**
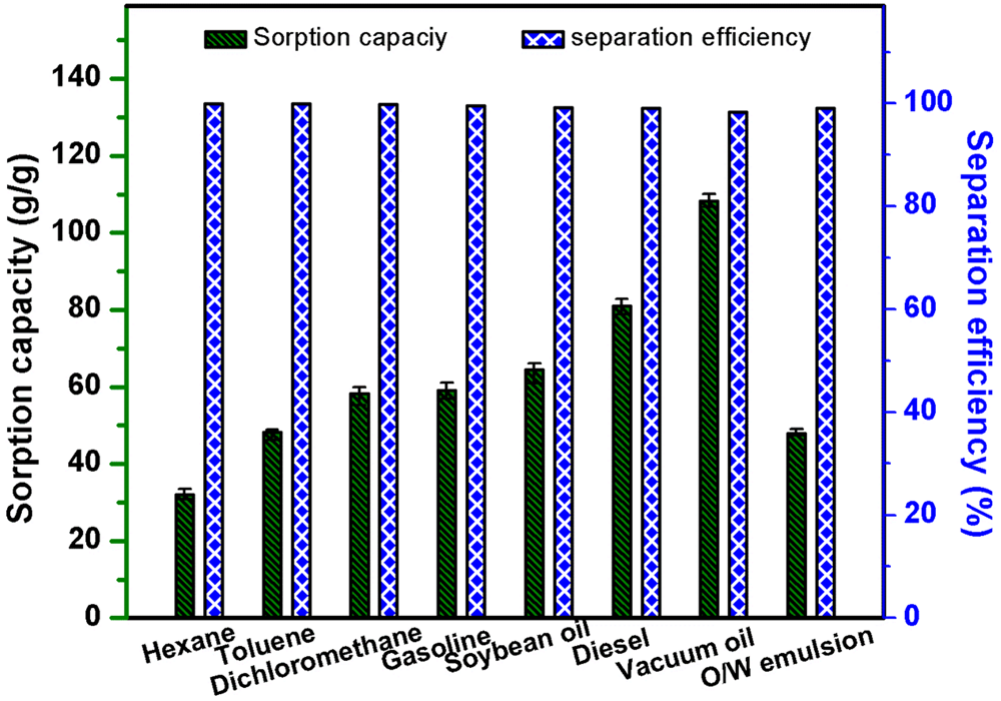
Oil absorption capacity and separation efficiency of the fabricated PU@ZnO@Fe_3_O_4_@SA sponge with different oils.

In the second experiment, which was conducted to determine the maximum oil absorption capacity (Fig. S6), the PU@ZnO@Fe_3_O_4_@SA sponge showed superior performance in absorbing all seven types of oil. The maximum oil absorbency of the fabricated sponge depended mainly on the properties (density and viscosity) of the oil (Fig. 8). For example, the absorbency of the sponge for hexane (density = 0.66 g/cm^3^) was 32.01 g/g compared to 80.98 g/g for diesel (density = 0.87 g/cm^3^). Furthermore, diesel has a much higher viscosity (103.90 mm^2^/s) than hexane (13.10 mm^2^/s), which can delay the wicking rate of oil from the sorbent surface and thus retain more oil in the porous structure of the sponge, leading to a higher k value. In the oil-water separation experiment (Fig. S7), the oil solution was absorbed and penetrated into the sponge, whereas the water layer remained above the PU@ZnO@Fe_3_O_4_@SA sponge, resulting in complete oil-water separation. The separation efficiencies of the fabricated sponge were 99.89%, 99.88%, 99.87%, 99.5%, 99.2%, 99.0% and 98.21% for hexane, toluene, dichloromethane, gasoline, soybean oil, diesel engine oil and vacuum pump oil, respectively (Fig. 8). The oil absorption efficiency results, which ranged from 32.1 to 108.9, are much better than those previously reported for sponge materials (Table 1)^22,39–42^. Separation of a toluene-in-water emulsion was also carried out *via* the same method. The sponge was wetted with oil when the water gradually separated from the emulsion (Fig. S8). The concentration of oil in the water before and after the separation was calculated from UV-Vis spectroscopy measurements. It was shown that the concentration of toluene in water decreased from 5.0% to 0.05% after the separation process, corresponding to separation of 99% of the oil from the emulsion.

**Table 1:** Comparison of the oil absorption capacity by sponge-based absorption materials between studies.

Material	Fabrication method	Types of absorbed oil/organic solvent	Max. oil abs. cap.	Ref.
PU-IP-PA sponge	Interfacial polymerization and molecular self-assembly	Crude oil, soybean oil, dichloromethane, compressor oil, diesel oil, n-hexane	16.5 ~ 29.9	^22^
Copper-C11H23COOAg-modified PU sponge	Solution-immersion processes	Lubricating oil, octane, decane, dodecane	13~19	^43^
Alkyl-chain-capped TiO_2_ with melamine sponge	Dipping	Methanol, ethanol, hexane, DMSO, DMF, acetone, chloroform, pump oil, motor oil	37.2 ~ 88.1	^44^
Lauryl methacrylate-modified PU sponge	Emulsifier-free emulsion polymerization and immersion	Diesel, kerosene	55.1 ~ 69.47	^45^
Mg–Al porous fiber/PU foam	Biotemplate method and foaming technology	Chloroform, soybean oil	25~43	^40^
CNT/PDMS-coated PU sponge.	Dip-coating	Soybean oil, motor oil, diesel, hexadecane, gasoline, hexane	15~25	^21^
Carbon soot-modified melamine sponge	Combustion flame process and dip coating	4-methyl-2-pentanone, cyclohexane, methanol, ethanol, hexane, toluene, crude oil, soybean oil, engine oil, pump oil	25~75	^46^
PU@PD@Ag@dodecylmercaptan sponge	Immersion	Diesel, petrol, crude oil, soybean oil, alcohol, hexane, acetone, toluene	18~45	^47^
PU@Fe_3_O_4_@SiO_2_@fluoropolymer sponge	Immersion	Petrol, toluene, chloroform	17~23	^48^
Melamine-lignin	Dip adsorbing	Peanut oil, sunflower oil. dodecane, hexadecane, gasoline, toluene, dichloromethane, hexane, styrene and chloroform	110~140	^49^
**PU@ZnO@Fe**_**3**_ **O**_**4**_ **@SA sponge**	**Microwave and dip-coating method**	**Hexane, vacuum pump oil, diesel engine oil, soybean oil**	**32.1 ~ 108.9**	**This study**

Comparison of oil absorption capacity between studies using absorption materials based on sponge form.

### Durability and recyclability

**Figure 9:**
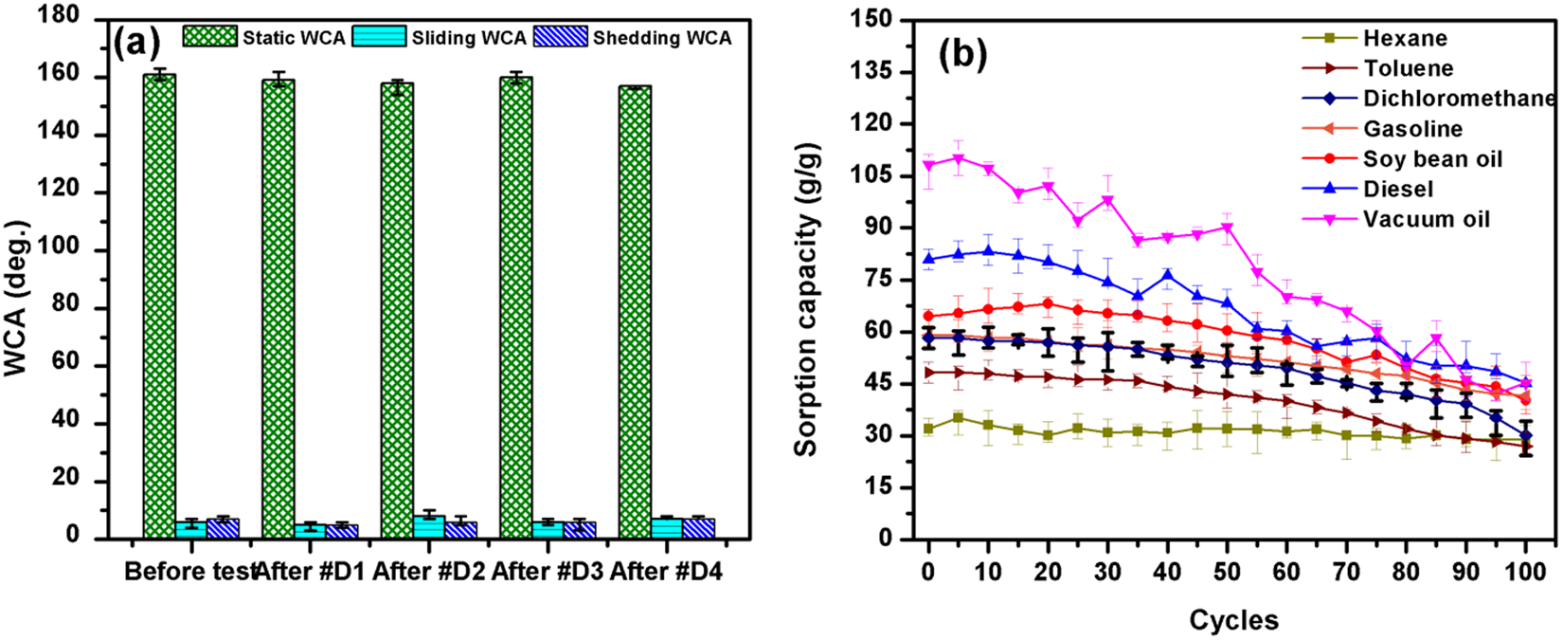
Stability and reusability of the PU@ZnO@Fe_3_O_4_@SA sponge for oil-water separation.

To estimate the durability of the fabricated sponge, an ultrasonic rinse test (D1), an iterative abrasion test (D2) and a wringing out by hand test (D3) were performed. The configuration of the setup for these tests is presented in Fig. S9, and the results are shown in Fig. 9a. The superhydrophobic PU sponge retained the original shape after the durability test, and the WCA was not significantly changed after a 30 min ultrasonic rinse test and drying step (D1), an abrasion test with 2000 gr loading (D2), a wringing out by hand (D3) test and a compression test (D4). These results confirmed that the fabricated PU@ZnO@Fe_3_O_4_@SA sponge is robust and stable, which is advantageous for real applications.

After oil absorption, the contaminated sponge was cleaned and recycled (Fig. 9b). After 100 separation/recovery cycles, the fabricated sponge still exhibited good reusability. The sorption capacity was nearly stable after 100 cycles for hexane and after the first 50 cycles for the other 3 oils. In the last 50 cycles, a decrease in the oil absorption capacity was unavoidable. This decrease was due to the presence of residual oil in the pores of the sponge that could not be totally removed by manual squeezing, particularly after many cycles.

## Conclusion

In this work, we successfully fabricated a durable, magnetic and superhydrophobic PU@ZnO@Fe_3_O_4_@SA sponge by a novel, facile and environmentally friendly method. By mimicking nature, a PU sponge was modified with ZnO, SA and Fe_3_O_4_ to provide the necessary high roughness, low surface energy and magnetic responsiveness, respectively. As a result, the fabricated sponge showed a very high static WCA (161°) and a very low dynamic WCA (sliding WCA = 7° and shedding WCA = 8°). The maximum sorption capacity of the fabricated sponge varied for the seven different oils examined (k = 32~108.9 g/g) due to the varying densities and viscosities of the oils, and these maximum sorption capacities were higher than those previously reported. The oil-water separation efficiency of the fabricated sponge exceeded 99%, and the absorbed oil could be easily recovered by simple mechanical squeezing. In addition, the sponge could be magnetically guided to the oil-polluted area and then quickly absorb the floating oil for efficient removal. Furthermore, the superhydrophobicity and oil absorbency of the fabricated sponge were maintained after stretching, compression, cleaning and repeated sorption cycles. The novel and superior performance of PU@ZnO@Fe_3_O_4_@SA makes the proposed sponge a promising candidate for the separation of oily pollutants from water and the cleanup of oil spills.

## Methods

### Materials and preparation of the superhydrophobic sponge

Commercial PU sponges - a product of Clean Life Co., Ltd., Korea (product No. 48475) - were purchased from a local store in Ulsan, Korea. Zinc acetate (Zn(CH_3_COO)_2_), ammonia solution (NH_4_OH), iron (II) chloride tetrahydrate (FeCl_2_.4H_2_O), anhydrous iron (III) chloride (FeCl_3_) and SA (C_18_H_36_O_2_) were purchased from Daejung Chemicals & Metals Co., Ltd., Korea. Extra-pure grade ethanol and acetone were purchased from Samchun Chemical Co., Ltd., Korea. N-hexane, vacuum pump oil, diesel engine oil, soybean oil, toluene, dichloromethane and gasoline were used to test the oil absorption capacity of the fabricated sponge samples. Characteristic information about the oils and organic solvents was taken from the catalog published by the manufacturers and is summarized in Table 2.

**Table 2:** List of the oils and organic solvents used.

Type	Manufacturer	Specification	Density (kg/L, 15 °C)	Viscosity (mm^2^/s, 40 °C)
Hexane	Samchun Chemical (Korea)	Pure 99.99%	0.66	13.10
Vacuum pump oil	Moresco Corporation (Japan)	NEOVAC MR-200	0.89	71.0
Diesel engine oil	GS Oil (Korea)	KIXX HD1 Cl-4/SL 15W-40	0.87	103.90
Soybean oil	Ottogi Ltd. (Korea)	Soybean oil extract	0.73	56.3
Toluene (methylbenzene)	Daejung Chemicals and Metals	Above 99.5%	0.8667	0.59
Dichloromethane	Daejung Chemicals and Metals	Above 99.5%	1.318	0.43
Gasoline	SK energy	Commercial product	0.77	0.673

**Figure 10:**
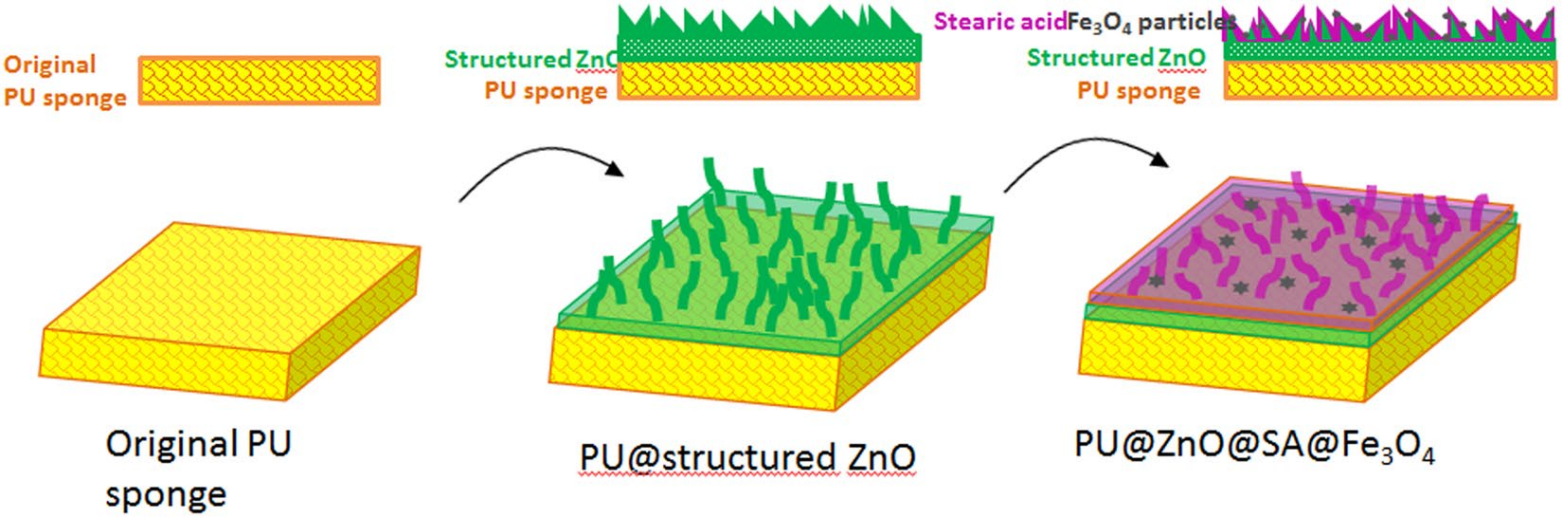
Illustration of the fabrication process of PU@ZnO@Fe_3_O_4_@SA.

The PU@ZnO@Fe_3_O_4_@SA sponge was prepared in two steps, as shown in Fig. 10.

#### PU sponge pretreatment

The original PU sponge was cut into cubes of the desired size (3 × 3 × 3 cm^3^) and cleaned with deionized (DI) water and ethanol several times to remove impurities that could cause unwanted reactions on the PU surface. The cleaned PU sponges were oven dried at 50 °C for 6 h, and this drying process did not affect the structure or the properties of the PU sponge. After the pretreatment, the coating steps were carried out according to the two fabrication processes described below.

#### Fabrication of ZnO flakes on the PU sponge surface

ZnO flakes were grown on the PU surface by a rapid microwave method^25^. A seeding solution of Zn^2+^ was prepared by dropwise addition of 25% NH_4_OH into 100 ml of 0.1 M zinc acetate, and a white precipitate was observed in the solution within a few seconds. Ammonia was added dropwise to the zinc acetate solution until the solution became transparent and reached a pH of pH 10–11. After that, the cubic PU sponges were immersed in a reaction flask containing the seeding solution with slight stirring. After 6 h, the reaction flask was heated in a commercial microwave oven (Daewoo KR-G20EW, 1120 W, 2450 MHz) in three steps, each consisting of 60 s of heating followed by 30 s of standing. Subsequently, the reaction flask was cooled to room temperature (approximately 20 °C) for 5 min. The PU sponges with a ZnO coating layer (PU@ZnO sponge) were taken out, rinsed with distilled water several times, and oven dried at 50 °C for 6 h.

#### Fabrication of PU@ZnO@Fe_3_O_4_@SA

Fe_3_O_4_ was synthesized by a co-precipitation method. After 100 ml of 0.1 M FeCl_3_ was added into 50 ml of 0.1 M FeCl_2_, chemical precipitation was achieved by adding an NH_4_OH solution under vigorous stirring. The reaction system was kept at 80 °C and a solution pH of 11–12 for 3 h. After the reaction system was cooled, the precipitates were separated by filtration and further washed with water and acetone until a neutral pH was reached. After that, the Fe_3_O_4_ product was oven dried at 60 °C for 12 h and milled to a powder using a manual grinder.

After 0.1 g of the prepared Fe_3_O_4_ powder was diluted in 100 ml of ethanol, the mixture was sonicated for 1 h at 30 °C. Then, the PU@ZnO sponge was soaked in the Fe_3_O_4_-ethanol solution for 60 min to obtain PU@ZnO@Fe_3_O_4_. The PU@ZnO sponge was uniformly coated with Fe_3_O_4_ particles. However, it was found that the interaction between the sponge and the Fe_3_O_4_ particles was weak. Finally, SA was coated on the sponge surface by immersing the PU@ZnO@Fe_3_O_4_ sponge in 50 ml of 10 mmol/L SA dissolved in ethanol for 3 h. This SA layer was used as the top coating layer to decrease the loss of Fe_3_O_4_ particles and thus maintain the stability of the material as well as provide the necessary chemical bonding for promoting superhydrophobicity. The PU@ZnO@Fe_3_O_4_@SA samples were finally washed with ethanol and distilled water to remove any excess reactants and then oven dried at 50 °C for 6 h.

### Characterization

Scanning electron microscopy (SEM) analysis was performed on an FE-SEM JEOL 6500 instrument to observe the morphology of the fabricated materials. A mapping technique combined with SEM analysis was used for elemental distribution analysis. The crystalline phases in the samples were determined by using an X-ray diffractometer (XRD, Bruker, Model AXS D8 ADVANCE). The XRD data were collected using Cu-Kα (λ = 0.154060 nm) radiation (step size, 0.02°; 2θ angular range = 10°–80°). The chemical composition and functional groups on the material surface were determined by X-ray photoelectron spectroscopy (XPS, Thermo Fisher Scientific, Model ESCALAB 250 XI XPS) and Fourier transform infrared spectroscopy (FT-IR, Varian 670/620). The magnetic properties of the materials were measured using a vibrating sample magnetometer (VSM PPMS Quantum Design, Inc.).

The WCA was measured using a contact angle meter (SmartDrop, Femtofab Co. Ltd., Korea) maintained by a computer-controlled device on an anti-vibration table and cabinet for reliable measurement. The volumes of the droplets used for static WCA and dynamic WCA measurements were 5 and 10 µl, respectively. In addition, the WCA tests were conducted at least five times at different positions on each sample. The sliding WCAs and shedding WCAs at intervals of 1° were also measured using a previously reported experimental unit^15^.

### Oil-water separation experiments

To demonstrate the oil absorption ability of the fabricated sponges, seven organic solvents and oils (including hexane, toluene, dichloromethane, gasoline, soybean oil, diesel engine oil and vacuum pump oil, which were dyed with oil red O dye) were used as sorbates. PU@ZnO@Fe_3_O_4_@SA sponges were applied in two experiments: (i) selective absorption of floating oil/organic solvent on water, (ii) separation of oil/organic solvent in a mixture with water and separation of emulsified oil in water.

The first experiment was carried out as shown in Fig. S5. After 10 ml of DI water was placed in a Petri dish, several drops of vacuum oil were added by a pipette, and the oil drops floated on the water surface. A cube of fabricated sponge (1 × 0.5 × 0.5 cm^3^) was placed in the Petri dish, and a magnet was used to guide the sponge to the locations of the oil drops. The resulting phenomenon was recorded by a digital camera. The sponge was then removed from the disk, and the purity of the remaining solution after oil absorption by the fabricated sponge was checked by UV-Vis spectroscopy.

The second experiment consisted of two different tests: determination of the maximum oil absorption capacity (Fig. S6) and the oil-water separation efficiency (Fig. S7). Stainless-steel mesh - as a sponge holder - was fixed on a homemade tube, and then a piece of PU@ZnO@Fe_3_O_4_@SA sponge (diameter of 7 cm and height of 2 cm) was placed on the mesh in the tube. An immiscible oil-water mixture and a toluene-in-water emulsion were used for the separation process. The toluene-in-water emulsion was prepared with a volume ratio between oil and water of 5% *via* the addition of 3 mg/mL of surfactant Tween80. The emulsion was stirred for 3 h and kept stable at room temperature for 7 days. For the maximum absorption capacity analysis (Fig. S6), oil drops were slowly dropped onto the sponge, and the oil was absorbed into the sponge until it was saturated with oil. The oil-saturated sorption state of the sponge was obtained when the sponge was completely covered with oil and no oil droplets fell onto the collecting disk underneath. The maximum oil absorption capacity (k) of the sponge was calculated by the weight-gain ratio, as shown in equation (1):

1 $${\rm{k}}=\frac{{W}_{a}-{W}_{b}}{{W}_{b}}({\rm{g}}/{\rm{g}})$$

where *W*_*a*_ is the weight of the sponge in the oil-saturated state and *W*_*b*_ is the weight of the sponge in the initial state.

For the separation efficiency analysis (Fig. S7), a mixture of oil and water (50%, v/v) or emulsified oil in water in a beaker was poured directly into the sponge surface, and another beaker was placed underneath to collect the excess oil after saturation. The water was repelled from the sponge surface while the oil was absorbed into the sponge. Then, the excess oil after saturation fell into the collection beaker underneath. After the full volume of the oil-water mixture was poured onto the sponge, the remaining water layer (oil free as determined by UV-Vis spectroscopy) on the sponge surface was collected merely by pouring into another beaker for volume measurement. The repellent ratio of water or oil-water separation efficiency was determined according to equation (2):

2 $${\rm{Separation}}\,{\rm{efficiency}}=\frac{{V}_{a}}{{V}_{b}}\times 100 \% $$

where *V*_*a*_ is the volume of the collected water remaining on the sponge surface after pouring the oil-water mixture and *V*_*b*_ is the volume of water in the initial mixture with oil before the mixture was poured onto the sponge.

### Durability and recyclability tests

The mechanical stability of the material plays an important role in real applications. Thus, mechanical stability tests were carried out on the PU@ZnO@Fe_3_O_4_@SA sponge. An ultrasonic rinse test (D1), an abrasion test with 1000-grit mesh sandpaper (D2), a wringing out by hand test (D3) and a compression test with a stress level of 0.000600 MPa at 80% strain (D4) were carried out to test the adhesion between the coated materials and the PU sponge. The details and digital images of these experiments are presented in Fig. S9.

The recyclability of the PU@ZnO@Fe_3_O_4_@SA sponge was tested by a simple method. After oil-water separation, the contaminated sponge was squeezed and rinsed with alcohol and water to remove the absorbed oil. Subsequently, the cleaned sponge was oven dried at 50 °C for 12 h and then used for the next 100 cycles.

## Electronic supplementary material

Supplementary information

Video S1

Video S2

Video S3

Video S4
