# Supplementary material for: Novel fabrication of a robust superhydrophobic PU@ZnO@Fe3O4@SA sponge and its application in oil-water separations
Source: Sci Rep. 2017 Dec 13;7:17520. doi: 10.1038/s41598-017-17761-9 (PMC5727532; doi:10.1038/s41598-017-17761-9)
Supplement: Supplementary file 1 — Supplementary information [file 41598_2017_17761_MOESM1_ESM.doc]

Supplementary Materials for

**Novel fabrication of a robust superhydrophobic PU@ZnO@Fe_3_O_4_@SA sponge and its application in oil-water separations**

Viet Ha-Tran Thi, Byeong-Kyu Lee*

Department of Civil and Environmental Engineering, University of Ulsan, Nam-gu,

Daehak-ro 93, Ulsan 680-749, Republic of Korea

* Correspondence to: bklee@ulsan.ac.kr

Tel: 82-52-259-2864, Fax: 82-52-259-2629

**This file includes:**

Figs. S1 to S9

Captions for Movies S1 to S4

**Other Supplementary Materials for this manuscript includes the following:**

Video S1 to S4


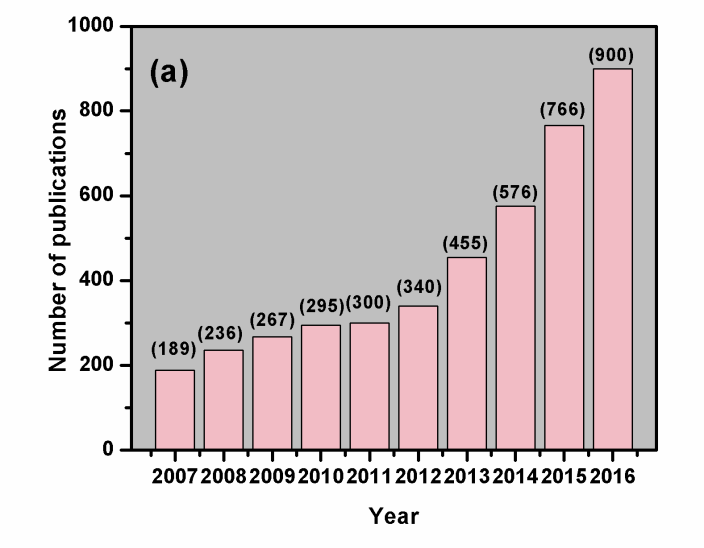

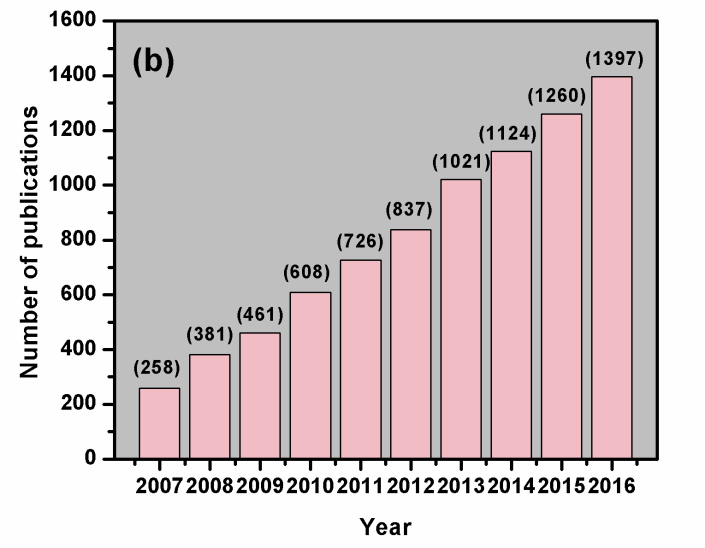


Fig. S1.

Number of paper published from 2007-2016 under the topic (a) oil-water separation and (b) superhydrophobic/superhydrophobicity (Source: ISI Web of Science)


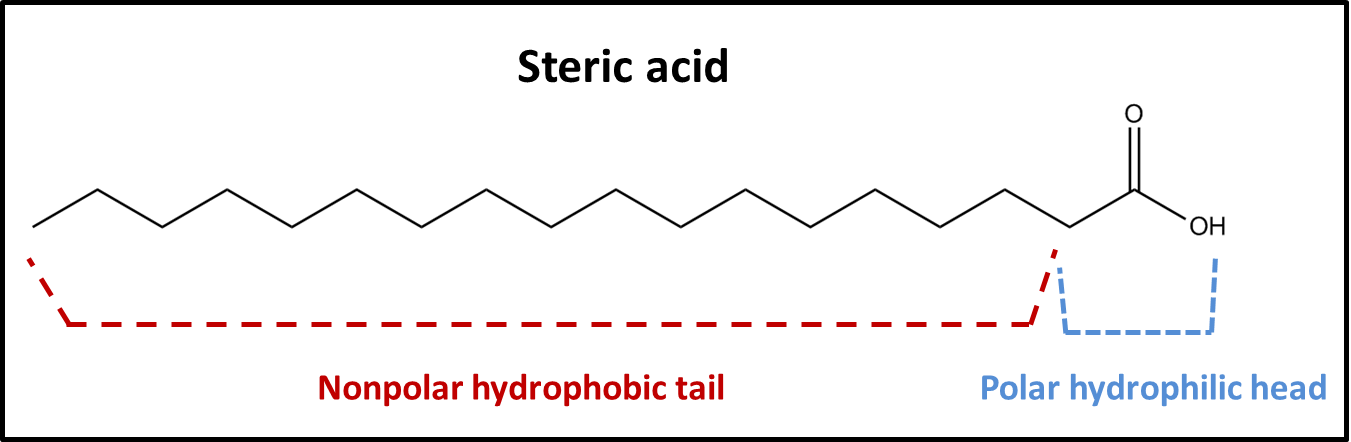


Fig. S2

Chemical structure of stearic acid


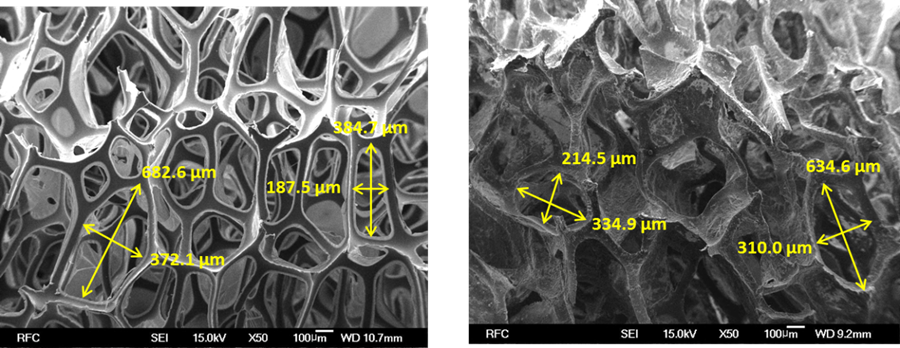


Fig. S3

SEM images of original PU and PU@ZnO@Fe3O4@SA sponge


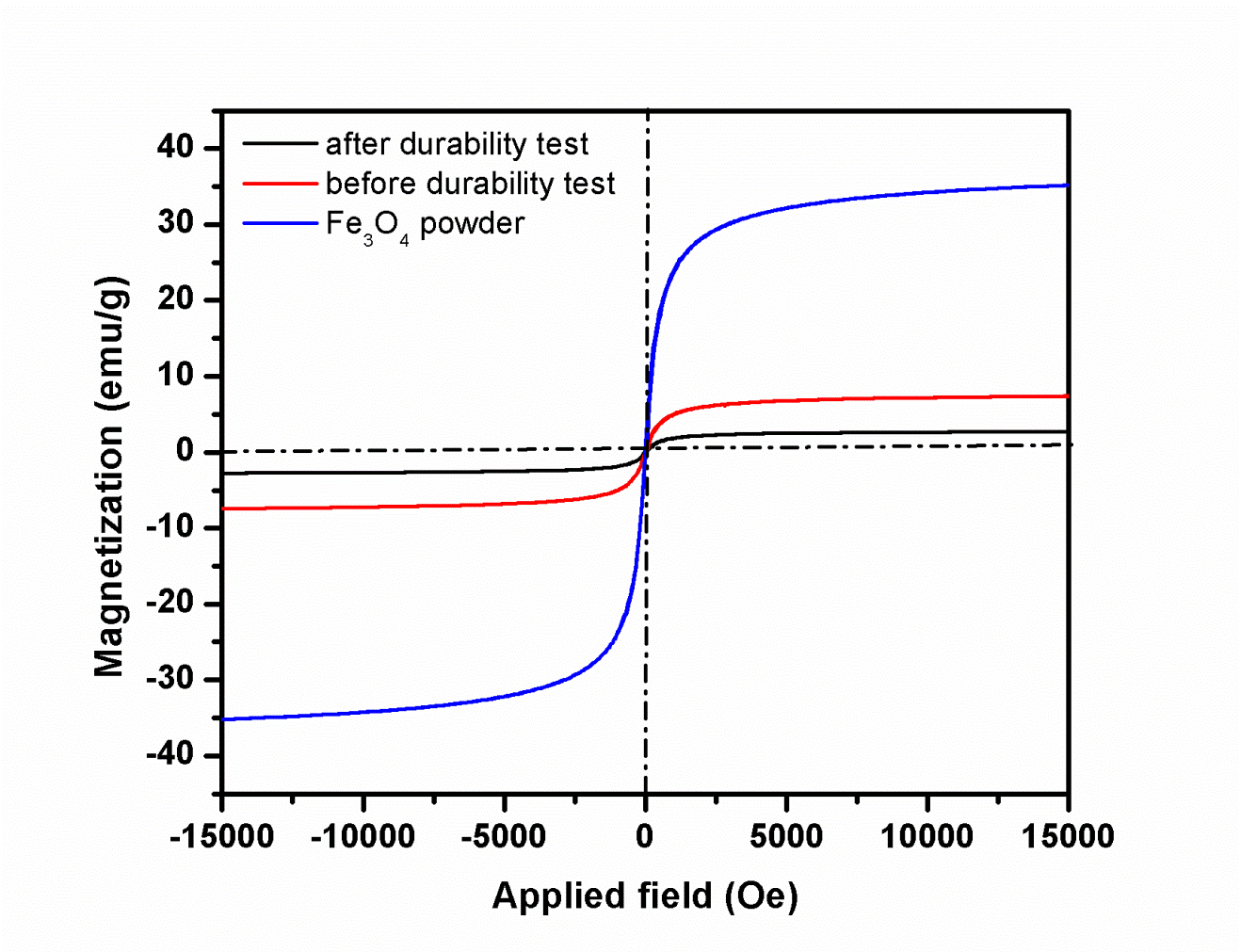


Fig. S4

Magnetization curves of Fe_3_O_4_ and sponge samples


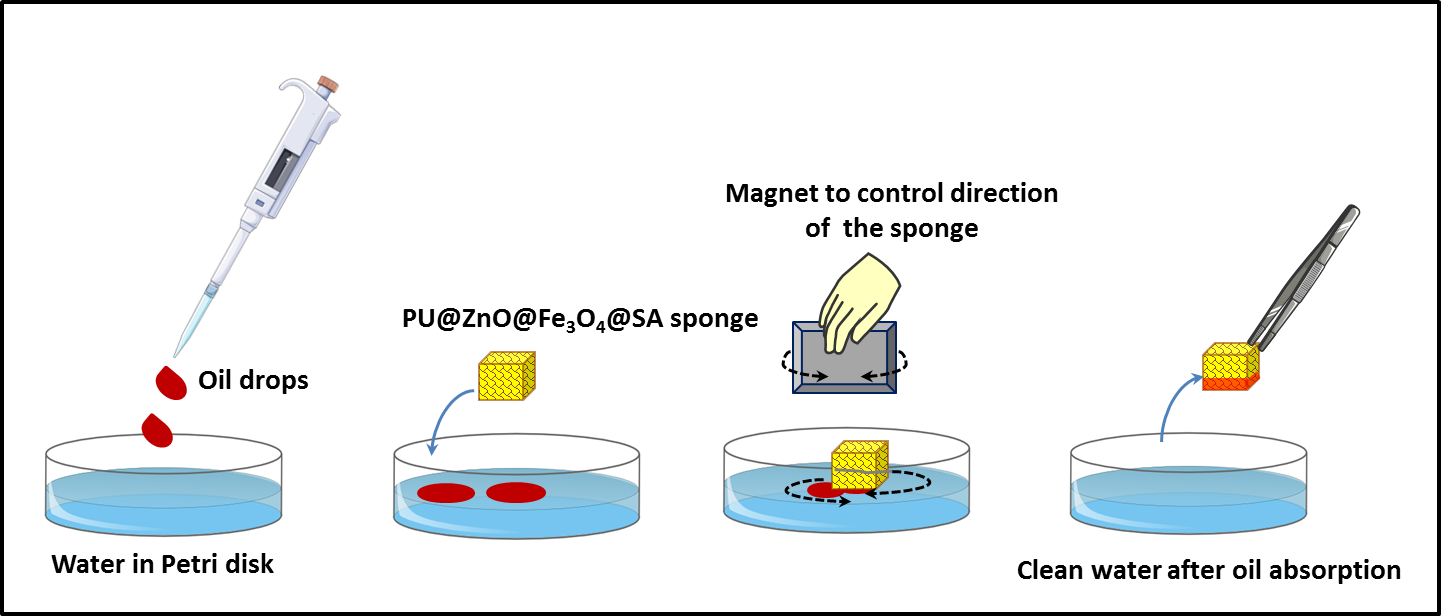


Fig. S5

Experiment set up for floating oil absorption with a magnet


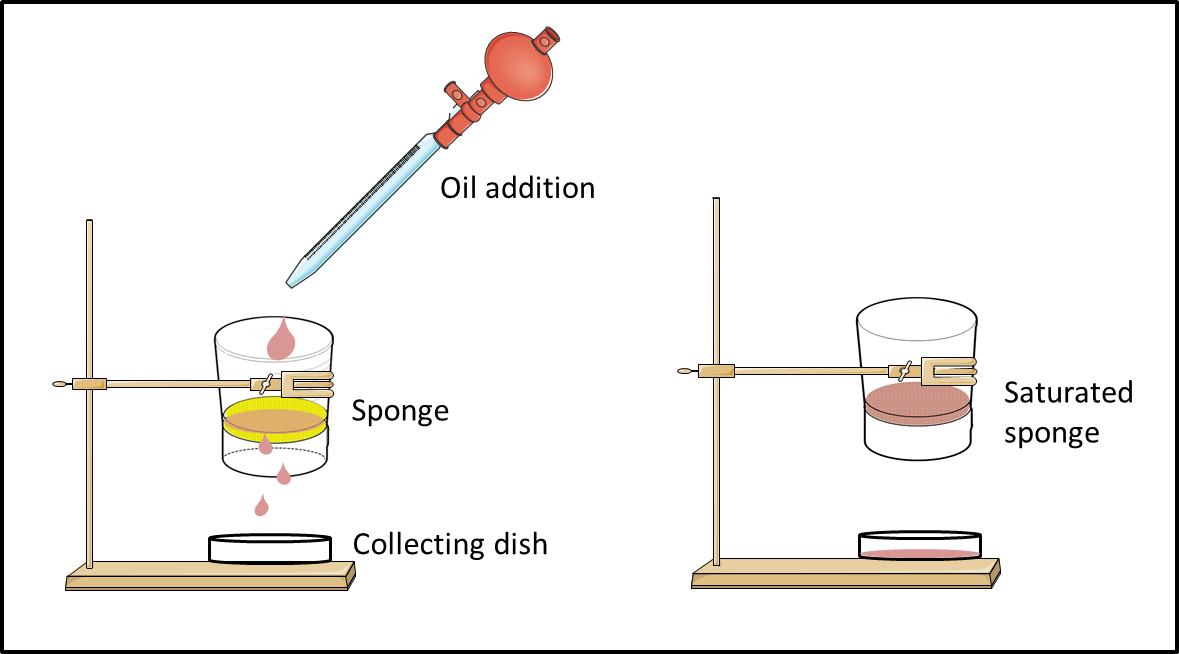


Fig. S6

Experiment set up used to determine the sponge’s maximum oil absorption capacity


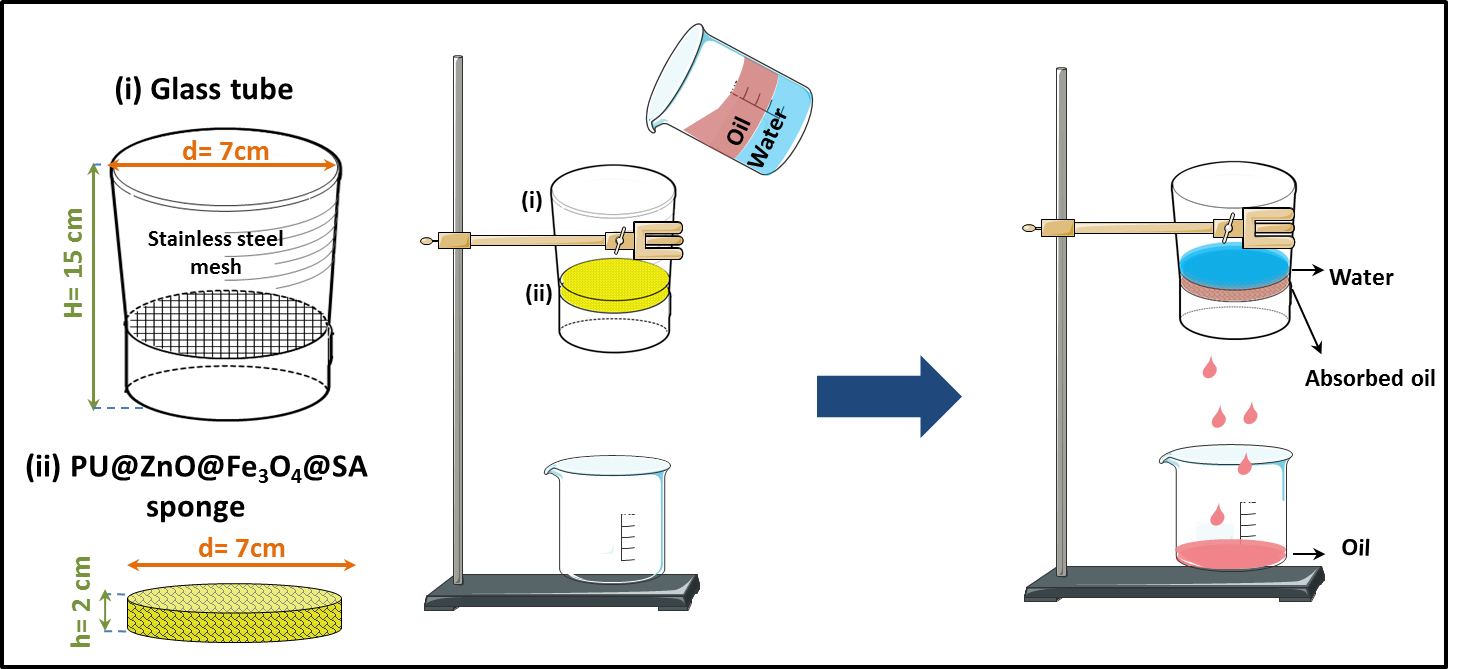


Fig. S7

Oil-water separation experiment design


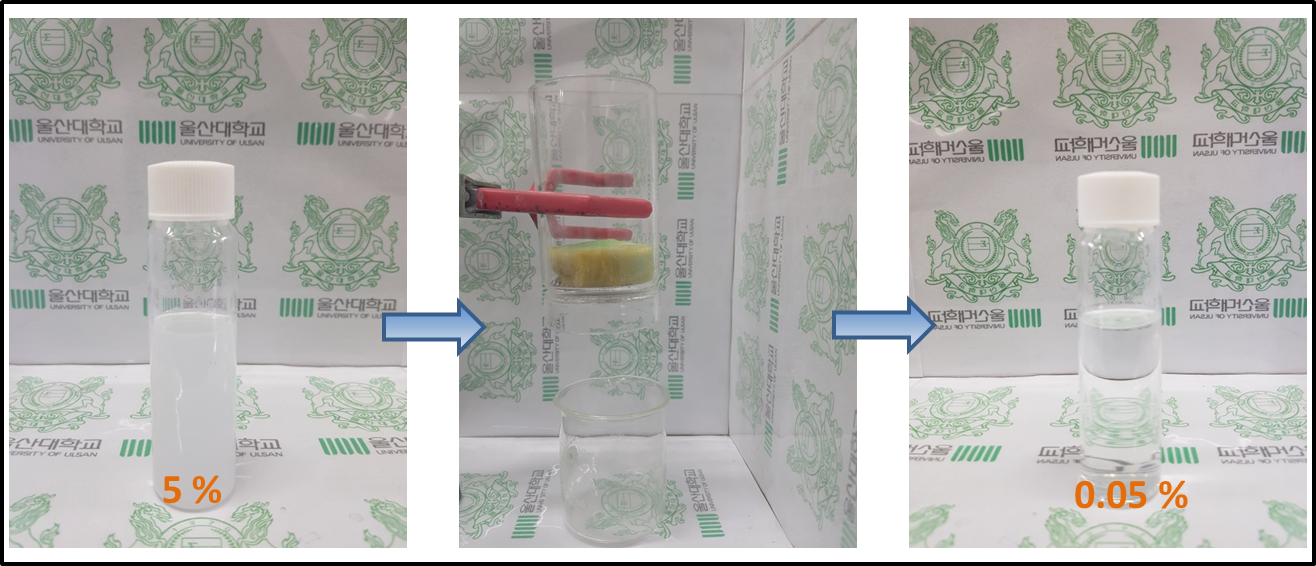

Fig. S8

Removal of emulsified oil using superhydrophobic sponge


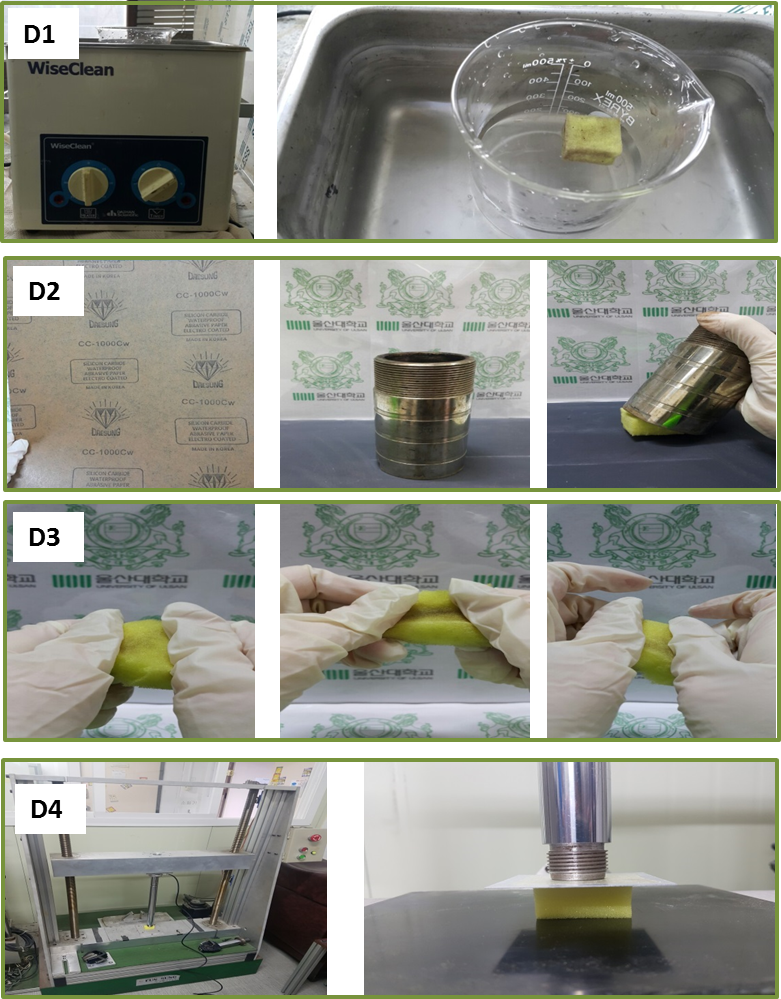


Fig. S9

Mechanical stability tests for PU@ZnO@Fe_3_O_4_@SA sponge samples

D1: PU@ZnO@Fe_3_O_4_@SA sponge was rinsed for 30 min in an ultrasonic bath, then dried at 50 ^o^C for 12 h

D2: Abrasion test was carried out for 50 times with 1000-grit mesh sandpaper, the weight of loading is 2000 g

D3: PU@ZnO@Fe_3_O_4_@SA sponge was twisted by hands in various directions for several times

D4: Compression test with stress level at 0.000600 MPa at 80 % strain

Video S1

Different wettability of original PU sponge and PU@ZnO@Fe_3_O_4_@SA sponge in dye solution

Video S2

Shedding angle at 8^o^ of PU@ZnO@Fe_3_O_4_@SA sponge

Video S3

Wettability with water and oil drops of sponges

Video S4

Floating oil absorption with the driven of magnetic
